# TRNT-1 Deficiency Is Associated with Loss of tRNA Integrity and Imbalance of Distinct Proteins

**DOI:** 10.3390/genes14051043

**Published:** 2023-05-05

**Authors:** Thet Fatica, Turaya Naas, Urszula Liwak, Hannah Slaa, Maryam Souaid, Brianna Frangione, Ribal Kattini, Antoine Gaudreau-Lapierre, Laura Trinkle-Mulcahy, Pranesh Chakraborty, Martin Holcik

**Affiliations:** 1 Department of Health Sciences, Carleton University, Ottawa, ON K1S 5B6, Canada; 2 Children’s Hospital of Eastern Ontario Research Institute, Ottawa, ON K1H 8L1, Canada; 3 Department of Cellular and Molecular Medicine, University of Ottawa, Ottawa, ON K1H 8M5, Canada

**Keywords:** SIFD, TRNT1, tiRNA, CCA adding, oxidative stress, SILAC

## Abstract

Mitochondrial diseases are a group of heterogeneous disorders caused by dysfunctional mitochondria. Interestingly, a large proportion of mitochondrial diseases are caused by defects in genes associated with tRNA metabolism. We recently discovered that partial loss-of-function mutations in tRNA Nucleotidyl Transferase 1 (*TRNT1*), the nuclear gene encoding the CCA-adding enzyme essential for modifying both nuclear and mitochondrial tRNAs, causes a multisystemic and clinically heterogenous disease termed SIFD (sideroblastic anemia with B-cell immunodeficiency, periodic fevers, and developmental delay; SIFD). However, it is not clear how mutations in a general and essential protein like TRNT1 cause disease with such clinically broad but unique symptomatology and tissue involvement. Using biochemical, cell, and mass spectrometry approaches, we demonstrate that *TRNT1* deficiency is associated with sensitivity to oxidative stress, which is due to exacerbated, angiogenin-dependent cleavage of tRNAs. Furthermore, reduced levels of TRNT1 lead to phosphorylation of Eukaryotic Translation Initiation Factor 2 Subunit Alpha (eIF2α), increased reactive oxygen species (ROS) production, and changes in the abundance of distinct proteins. Our data suggest that the observed variable SIFD phenotypes are likely due to dysregulation of tRNA maturation and abundance, which in turn negatively affects the translation of distinct proteins.

## 1. Introduction

The maturation of tRNAs (both mitochondrial and cytoplasmic) is a multi-step process that requires 5′ and 3′ processing, extensive base modifications, CCA addition, and aminoacylation. For cytoplasmic tRNAs, these steps occur in a precise order and are performed in distinct cellular compartments; in contrast, all mt tRNA processing steps occur in mitochondria [1]. The CCA addition is performed by a unique CCA-adding enzyme, tRNA nucleotidyltransferase (TRNT1 in humans or CCA1 in yeast). *TRNT1* encodes the only human CCA-adding enzyme, an RNA polymerase required for the post-transcriptional, template-independent addition of two cytosines and one adenosine to the 3′ end of both cytosolic and mitochondrial tRNAs [2]. This 3′ addition is required for aminoacylation, correct positioning on the ribosome, and subsequent protein synthesis [3]. Following the addition, the CCA trinucleotide acts as an anti-determinant for 3′ endoribonuclease activity [4]. The 3′ CCA tRNA terminus is subject to frequent cleavage or degradation, and TRNT1 also functions to maintain and repair previously added CCA sequences [5]. Importantly, TRNT1 can discriminate against tRNA backbone damage, keeping damaged tRNAs from incorporation into the translation machinery [6].

We have previously reported that partial loss-of-function mutations in *TRNT1* cause a novel disease termed SIFD (sideroblastic anemia with B-cell immunodeficiency, periodic fevers, and developmental delay; SIFD) [7]. Subsequent publications have revealed significant clinical heterogeneity of disease associated with TRNT1 deficiency. In a recent systematic review, 41 unique mutations have been noted in 58 individual cases [8]. SIFD is predominantly characterized by severe sideroblastic anemia (SA) or microcytic anemia and very early onset in the neonatal period or infancy with a median age of 4 months (range 0–252 months), although a broad range of additional variable clinical features is present [2,8].

Several clinical features of TRNT1 patients are consistent with ‘classical’ mitochondrial dysfunction (e.g., sideroblastic anemia, lactic acidosis, sensorineural hearing impairment), and we and others have shown impaired respiration and composition of the oxidative phosphorylation (OXPHOS) complexes in SIFD patients [9,10]. Other patient characteristics such as B-cell immunodeficiency and periodic fevers are not, however, generally associated with mitochondrial dysfunction, suggesting other cellular consequences of *TRNT1* deficiency play important roles in the pathobiology associated with *TRNT1* mutations. Patients with SIFD have been observed to deteriorate, often catastrophically, following inflammatory episodes and/or infections. Furthermore, the accumulation of ROS has long been postulated to be implicated in the pathobiology of mitochondrial diseases. We, therefore, hypothesized that *TRNT1*-deficient cells could have disordered stress responses, specifically to oxidative stress.

Oxidative stress is a phenomenon ascribed to an imbalance between the synthesis and accumulation of ROS and antioxidant defenses in the body that are responsible for detoxifying these free radicals [11,12]. Thus, an abundance of free radicals and oxidants, along with the inability of cells to clear them, generate oxidative stress, a harmful process that holds injurious effects on numerous important cellular structures, including deoxyribonucleic acid (DNA), proteins, lipoproteins, lipids, and membranes. As a result, oxidative stress can cause damage to cells and tissues, contributing to a variety of diseases over time [12].

In this study, we demonstrate that SIFD patient-derived fibroblasts are extremely sensitive to oxidative stress induced by H_2_O_2_ or arsenite treatment. We further show that this sensitivity is mediated by angiogenin and is due to the cleavage of cytoplasmic tRNAs. Consistent with this, acute reduction of TRNT1 by siRNA resulted in increased phosphorylation of eIF2α and elevated levels of mitochondrial ROS. Although phosphorylation of eIF2α is typically linked to general translational regulation, smaller changes in eIF2α phosphorylation (such as those seen in cells with reduced levels of TRNT1) lead to altered selective translation of specific mRNAs [13]. Indeed, a mass spectrometry experiment (SILAC) on SIFD patient and control cells identified changes in the abundance of distinct groups of proteins, and we have validated decreased expression of Calponin 2 in SIFD patient-derived cells and cells with acute reduction of *TRNT1*.

## 2. Materials and Methods

### 2.1. Cell Culture and Transfection

Control and patient fibroblasts, HEK 293T, or HeLa cells were maintained at 37 °C, 5% CO_2_ in Dulbecco’s Modified Eagle’s Medium (DMEM) (Thermo Scientific, Ottawa, ON, Canada) supplemented with 10% fetal calf serum, 2 mM L-glutamine, and 1% antibiotics (100 mg/mL streptomycin and 100 U/mL penicillin). Control fibroblasts were obtained from Coriell Institute (C1; Cat# GM08680, C4; Cat# GM00498). Patient fibroblasts with mutations in the *TRNT1* gene were previously described in [7]. For oxidative stress induction, the cells were treated with 0.25 mM of hydrogen peroxide solution (H_2_O_2_) (Sigma-Aldrich, Oakville, ON, Canada) or 50 μM Sodium Arsenite (NaAsO_2_) solution (Sigma-Aldrich) diluted in a medium for indicated times.

For TRNT1 or angiogenin knock-down, small interfering RNA (siRNA) transfections were performed using Lipofectamine RNAiMAX according to the protocol provided by the manufacturer (Invitrogen Life Technologies, Burlington, ON, Canada). Briefly, cells were seeded at a density of 75 × 10^3^ cells/well in 6-well plates or 5 × 10^3^ cells/well in 96-well plates. The cells were transfected 24 h later in antibiotic-free DMEM with a pool of four siGenome human angiogenin (40 nM) (Dharmacon, Cat # D-011206,-01, -02, -03, -04), or TRNT1 [7], or a non-silencing control siRNA (Dharmacon, Dharmacon, 5′-UUCUCCGAACGUGUCACGUdTdT-3). Cells were collected for analysis 72 h post-transfection.

### 2.2. Kinetic Measurement of Cytotoxicity and Caspase-3/7 Activation

Control and patient fibroblasts were treated with hydrogen peroxide or sodium arsenite as indicated above and incubated with either YOYO-1 dye (Invitrogen Life Technologies) or Incucyte Caspase-3/7 Green Dye (Sartorius, Oakville, ON, Canada), and the incubation was monitored for 48 h using the IncuCyte Zoom S3 Live-Cell Imaging System (Sartorius) as per the manufacturer’s instruction.

### 2.3. Lentiviral Vector Production and Cells Transduction

A lentiviral construct expressing wt human TRNT1 (pReceiver-LV216cFalg-SV40-mcherry-IRES-Puromycin) was purchased from Genecopeia (Cat #EX-Z5336-LV216). An empty construct (Cat #EX-NegLV216) was used as a negative control. Replication-defective lentiviral vectors were generated by transient transfection of 293T human kidney cells. Cells were seeded at 15 × 10^6^ cells in complete DMEM medium (Thermo Scientific) supplemented with 10% fetal calf serum, 2 mM L-glutamine, and 1% antibiotics (100 mg/mL streptomycin and 100 U/mL penicillin) (Invitrogen Life Technologies). The cells were incubated overnight, and then the media was replaced with DMEM+10% FCS without antibiotics, and the cells were transfected at 60–70% confluence using JetPrime transfection reagents (VWR International, Mississauga, ON, Canada). For each 15 cm plate, 20 μg of pReceiver-LV216cFalg-SV40-mcherry-IRES-Puromycin or an empty vector, and a 63 μg mixture of the trans-lentiviral packaging system (Thermo Fisher) including pTLA1-PAK, pTLA1-ENZ, pTLA1-ENV, pTLA1-TOFF, and pTLA1-Tat/Rev plasmids. Viral particle-containing supernatants were harvested 72 h post-transfection and centrifuged at 1600× *g* at 4 °C for 10 min to remove the cell debris, followed by filtration through a sterile 0.22–0.45 μm low protein binding filter (Millipore, Etobicoke, ON, Canada). The viral stocks were concentrated by adding 20% sucrose cushion and then ultra-centrifugation at 28,000 rpm for 2 h at 4 °C using Beckman SW32 Ti rotors. The viral pellets were suspended in PBS and stored at −80 °C until use. 

For transduction, HEK 293T cells or human control and patient fibroblasts were seeded on 6-well cell culture plates and incubated with the same titer of TRNT1-expressing or control lentivirus in the presence of 8 ug/mL polybrene. Seventy-two hours after infection, the number of the transduced cells (m-cherry positive cells) was determined by the IncuCyte Zoom imaging. Puromycine (1 ug/mL) was added to the media for the selection of stably transduced cells.

### 2.4. Quantitative Real-Time PCR

Total RNA was extracted using RNAzol RT (Sigma-Aldrich) and quantified using a Nanodrop 2000 Spectrophotometer (ThermoScientific). A total of 600 ng RNA was reverse transcribed (qScript™ cDNA Supermix, Quanta Biosciences, Beverly, MA, USA) to obtain cDNA (25 °C × 5 min, 42 °C × 30 min, 85 °C × 5 min, and hold at 4 °C). For quantitative real-time PCR, human angiogenin primers (Hs ANG 2 SG) (Qiagene, Toronto, ON, Canada, Cat# QT01675212) and GAPDH primers (Qiagene, Cat#QT01192646) were used with SYBR Green Fast mix (Quanta Bioscience) and samples were tested in triplicate with the following cycling conditions: 95 °C × 2 min, 95 °C × 10 s, 55 °C × 30 s, and 72 °C × 10 s for 40 cycles (Mastercycler^®^ ep realplex, Eppendorf, Mississauga, ON, Canada). Primer efficiencies were tested, and standard curves were generated for each primer set.

### 2.5. Western Blotting

Cells were washed in PBS and lysed in a RIPA buffer for 30 min at 4°C, followed by centrifugation at 12,000× *g* for 10 min to pellet debris. Protein concentrations were determined by Pierce BCA protein assay (Thermo Scientific, Cat. # 23225), and equal amounts of protein extract were separated by 10 % SDS-PAGE (or 14% for angiogenin) and transferred to the PVDF membrane. Membranes were blocked in a 5% milk-blocking buffer for 1 h at room temperature and then probed overnight at 4 °C with primary antibodies, washed, then probed for 1 h at room temperature with secondary antibodies. The following primary antibodies were used: anti-TRNT1 (Bio-Techne, Toronto, ON, Canada, NBP1-86589), anti-angiogenin (Abcam, Boston, MA, USA, Cat# ab10600), anti-α-tubulin (Abcam, Cat# ab7291), anti-GAPDH (Advanced ImmunoChemical, Long Beach, CA, USA, Cat# 06-V-G4-C5), anti-MIF (Sigma-Aldrich; HPA003868), anti-ECE1 (Origene, Burlington, ON, Canada; TA349913), anti-CNN2 (Sigma; HPA049095), anti-PTGIS (Acris Antibodies, Herford, Germany; AP14903PU-N), anti-IGFBP3 (Abcam; ab137370), anti-CNN1 (Millipore; ABT129), anti-FHL1 (Origene; TA335865), anti-MYL9 (Abcam; ab191393), anti-ALDH1A1 (Cell Signaling, Whitby, ON, Canada; 12035), anti-eIF2α (Abcam, ab5369), anti-eIF2α-P (S51) (Invitrogen Life Technologies, 44-728G). Anti-rabbit and anti-mouse IgG HRP-conjugated secondary antibodies (Cell Signaling) were used at 1:2000. The antibody complexes were detected using an ECL system (GE Biosciences, Piscataway, NJ, USA) and exposed onto HyBlot CL Autoradiography Film (Thermo Scientific) when developed using a Kodak X-OMAT 2000A processor or exposed the blots using ChemiDocTM MP (Bio-Rad, Mississauga, ON, Canada).

### 2.6. tRNA Cleavage

Normal human fibroblasts were seeded at a density of 900,000 cells per 10 cm dish, grown for 24 h, and treated with 50 μM NaAsO_2_ for indicated times. Total RNA was extracted using RNAzol RT (Sigma, R4533) following the manufacturer’s protocol. A total of 1 μg RNA was then separated using precast 15% Novex TBE-Urea gel (ThermoFisher, EC6885BOX), and the gels were stained with SYBR Gold (ThermoFisher, S33102) diluted 1:10,000 in 1× TBE for 30 min prior to imaging (BioRad). dsRNA ladder (NEB, Whitby, ON, Canada, NO363S) was used as a size marker. To assess tRNA cleavage following either TRNT1 or angiogenin knock-down, cells were reverse-transfected with appropriate siRNA (see above) for 48 h prior to NaAsO_2_ treatment.

### 2.7. ROS

Fibroblasts were cultured on coverslips for 24 h in DMEM and then loaded with a MitoSox reagent (Invitrogen, M36008) as per the manufacturer’s instructions. Cells were incubated at 37 °C in the dark for 10 min and then washed. Nuclei were stained with 1 μg/mL Hoechst 33342 (Sigma-Aldrich) for 5 min with gentle rocking. Coverslips were then rinsed three times for 5 min with PBS and subsequently mounted onto Superfrost Microscope Slides (25 × 75 × 1.0 mm, Thermo Scientific) using a Dako fluorescent mounting medium (Agilent, Mississauga, ON, Canada), then set aside in a dark area to dry before applying a sealant over the edges of the coverslips. Images were taken using the Olympus 1 × 81 confocal microscope using the 60× water-based objective.

### 2.8. Immunofluorescent Microscopy

Fibroblasts were cultured on coverslips or 96-well plates (Corning) for 24–72 h in DMEM media. Cells were treated according to the rescue protocol or left untreated. Cells were fixed with 3.7% formaldehyde at room temperature for 15 min, washed three times with PBS, and permeabilized for 15 min with 0.2% Triton X-100 (Sigma) in PBS. Cells were washed three times with PBS and blocked with 1% bovine serum albumin (Invitrogen) for 15 min. Cells were incubated for one hour at room temperature with anti-CNN2 (Sigma; HPA049095) and anti-FLAG (Cell Signaling; 8146) primary antibodies diluted 1/1000 in a TX-100/BSA buffer containing 0.2% BSA and 0.004% TX-100 in PBS. Cells were washed in PBS and incubated with AlexaFluor-488 anti-rabbit or AlexaFluor-594 anti-mouse antibodies for one hour, covered at room temperature. Cells were washed with PBS and stained with 1ug/mL Hoeschst33342 (Sigma). Cells were imaged using the Olympus Fluoview FV-1000 Laser Confocal Microscope.

### 2.9. Stable Isotope Labeling by Amino Acids in Culture (SILAC)-Based Liquid Chromatography Mass Spectrometry (LC-MS/MS) Analysis

Control and patient fibroblasts were differentially labeled by growth for at least seven passages in SILAC media containing either environmental forms of the essential amino acids arginine and lysine (Arg0Lys0, light media) or isotopic forms (Arg10Lys8, Heavy). For the first run, control cells were labeled in light media and patient cells in heavy media. For the second run, the labeling was flipped so that control cells were grown in heavy media and patient cells in light media. Equivalent total protein amounts (25 μg each) of control and patient extracts were combined and treated with DTT (10 mM, 95 °C, 10 min) to reduce proteins, and then iodoacetamide (50 mM, 21 °C, 30 min in the dark) to alkylate proteins. An LDS sample buffer was added, and the combined sample resolved in one lane on a NuPAGE 4–12% BisTris gel. The gel was stained using SimplyBlue Safestain (Thermo Fisher), and the entire lane was cut into 12 slices. Each slice was cut into 2 × 2 mm fragments, destained, and digested overnight at 30 °C with Trypsin Gold (ThermoFisher).

An aliquot of each tryptic digest was analyzed by LC-MS/MS on an Orbitrap Fusion Lumos system (ThermoScientific) coupled with a Dionex UltiMate 3000 RSLC nano HPL. The raw files were searched against the Human UniProt Database using MaxQuant software v1.5.5.1 (http://www.maxquant.org (accessed on 10 March 2023)) and the following criteria: peptide tolerance = 10 ppm, trypsin as the enzyme (two missed cleavages allowed), and carboxyamidomethylation of cysteine as a fixed modification. Variable modifications are the oxidation of methionine and N-terminal acetylation. Heavy SILAC labels were Arg10 (R10) and Lys8 (K8). Quantitation of SILAC ratios was based on the razor and unique peptides, and the minimum ratio count was 2. The peptide and protein FDR were 0.01. The heavy:light ratios reflected the relative amount of each protein in mitochondria isolated from control vs. patient fibroblasts.

### 2.10. Statistical Analysis

YOYO-1 and caspase 3/7 data collection and analysis were performed using the Essen IncuCyte Zoom S3 kinetic cell imager and the IncuCyte software v 2020B. Densitometry of immunoblots and RNA gels was analyzed using LI-COR^®^ Image Studio software and BioRad^®^ Image Lab software v 6.1.0 build 7. Where appropriate, data were analyzed using a one-tailed unpaired Student t-test using the GraphPad Prism software v 7.01 and are expressed as a mean ± standard deviation of at least three independent experiments.

## 3. Results

### 3.1. TRNT1-Deficient Fibroblasts Are Sensitive to Oxidative Stress

**Figure 1:**
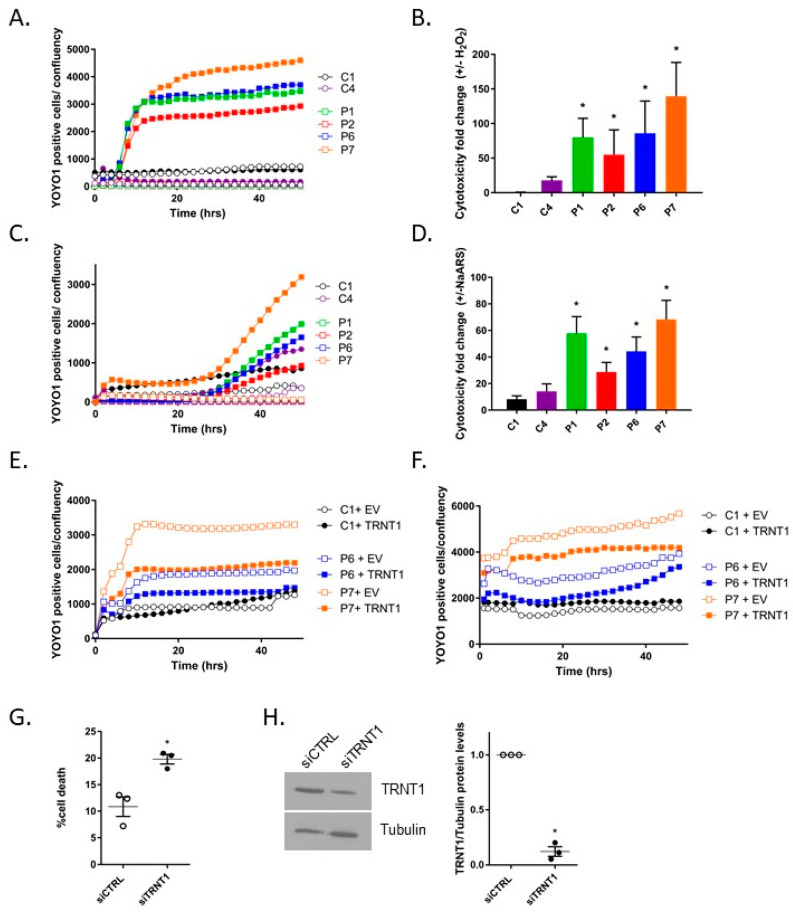
TRNT1 is the determinant of cellular sensitivity to oxidative stress. (**A**–**D**) Normal (C1 and C4) and patient-derived fibroblasts (P1, P2, P6, P7) were treated with 0.25 mM H_2_O_2_ (**A**,**B**) or 50 μM NaAsO_2_ (**C**,**D**). The number of dead cells was monitored using the IncuCyte Live Imaging System and a DNA-binding fluorescent reagent YOYO-1 over time (**A**,**C**). The cytotoxicity fold change in H_2_O_2_ or NaAsO_2_ treated cells (solid symbols) relative to non-treated cells (empty symbols) was determined at 48 h for three independent experiments ((**B**,**D**); *t*-test, * *p* < 0.05). (**E**,**F**) Normal (C1) and patient-derived fibroblasts (P6 and P7) were transduced using a lentivirus vector expressing TRNT1 or empty control, subsequently treated with either 0.25 mM H_2_O_2_ (**E**) or 50 μM NaAsO_2_ (**F**) and monitored using the IncuCyte Live Imaging System and YOYO-1 dye. (**G**,**H**) Control (C1) fibroblasts were transfected with siCTRL or siTRNT1 for 72 h, subsequently treated with 50 μM NaAsO_2_ for 48 h, and cell viability was determined using YOYO-1 dye. The percentage of cell death was calculated relative to untreated cells ((**G**); *n* = 3, *t*-test, * *p* < 0.05). The relative levels of TRNT1 and Tubulin (loading control) were determined by western blotting (**H**). A representative blot is shown on the left; a densitometric analysis of replicates is shown on the right (*n* = 3, *t*-test, * *p* < 0.05).

To examine the effect of *TRNT1* mutations on the cellular response to oxidative stress, two control, and four SIFD patient-derived fibroblasts were treated with hydrogen peroxide (H_2_O_2_) or sodium arsenite (NaAsO_2_) to induce oxidative stress. The cytotoxicity of the treatment was determined using YOYO1, a DNA-binding fluorescent dye, and was monitored for 48 h using the IncuCyte Live Imaging System (Figure 1A–D). When compared to normal fibroblasts, the SIFD patient-derived fibroblasts exhibited significantly higher sensitivity to oxidative stress induced by either H_2_O_2_ (Figure 1A,B) or NaAsO_2_ (Figure 1C,D). In addition, since numerous studies linked oxidative stress to the induction of apoptosis [14], we also measured the induction of apoptosis in control and SIFD patient-derived fibroblasts. When compared to normal fibroblasts, the SIFD patient-derived fibroblasts exhibited significantly higher caspase3/7 activation in response to oxidative stress induced by either H_2_O_2_ or NaAsO_2_ (Supplementary Figure S1). To determine if the observed enhanced sensitivity of SIFD patient-derived cells to oxidative stress was due to the mutations in *TRNT1*, we transduced C1, P6, and P7 cells with a lentivirus vector expressing either wild-type TRNT1 or empty control. The transduction efficiency was determined by the mCherry fluorescent signal, while the levels of TRNT1 protein expression were detected by western blotting (Supplementary Figure S2). The transduced control and SIFD patient-derived cells were subsequently treated with H_2_O_2_ (Figure 1E) or NaAsO_2_ (Figure 1F), and cytotoxicity was monitored over time. We observed that expression of TRNT1 in SIFD patient-derived fibroblast partially rescued both H_2_O_2_ and NaAsO_2_-induced cytotoxicity.

In a converse experiment, we used siRNA to reduce levels of TRNT1 in C1 control fibroblasts. Cells were subsequently treated with NaAsO_2_ for 48 h, and cytotoxicity was measured using YOYO-1 dye. We observed that reducing levels of TRNT1 significantly increased cells’ sensitivity to oxidative stress induced by NaAsO_2_.

### 3.2. Oxidative Stress Induced tRNA Cleavage in a TRNT1-Dependent Manner

**Figure 2:**
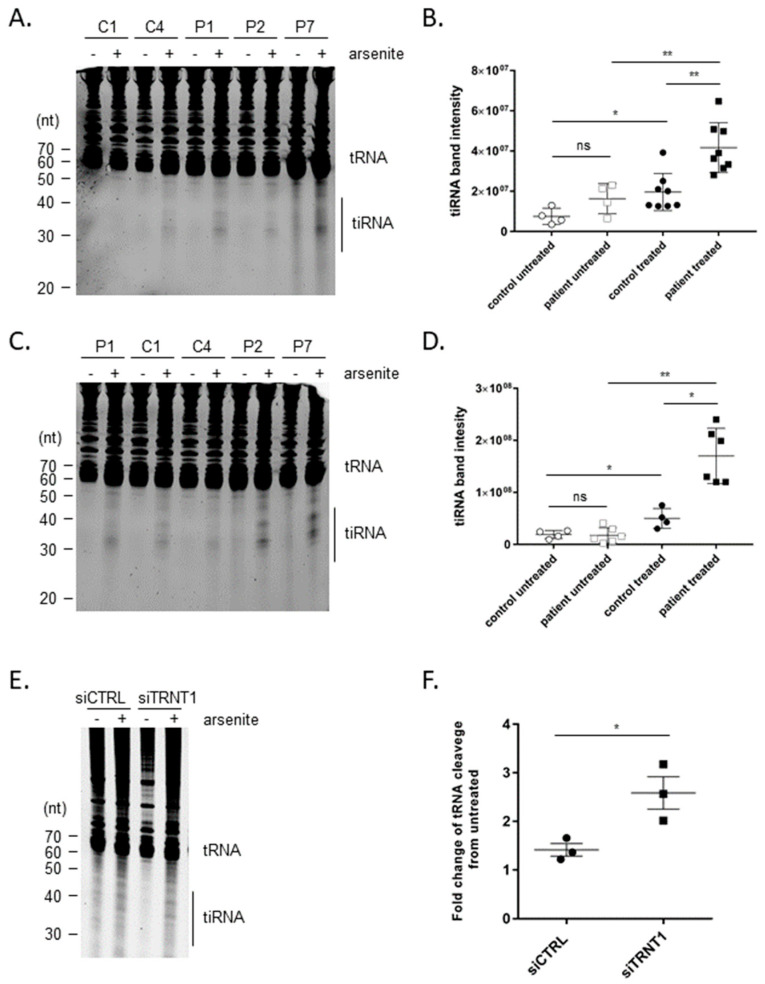
tRNA cleavage induced by oxidative stress is exacerbated in TRNT1 patient-derived cells. Normal (C1 and C4) and patient-derived fibroblasts (P1, P2, P7) were treated with 50 μM NaAsO_2_ for 1.5 h (**A**) or 18 h (**C**), and total RNA was extracted and separated by gel electrophoresis as described in the Materials and Methods. Representative images are shown. The intensity of the cleaved tRNA bands (tiRNA) for each group is shown in (**B**,**D**). The expression of cleaved tRNA was quantified in two independent experiments. For statistical analysis, control and patient cells were pooled together (*t*-test, * *p* < 0.05, ** *p* < 0.01, ns—not significant). Normal (C4) fibroblasts were transfected with siTRNT1 or siCTRL and subsequently treated with 50 μM NaAsO_2_ for 18 h (**E**). Total RNA was extracted and separated by gel electrophoresis as described in the Materials and Methods. The representative image is shown. The intensity of the cleaved tRNA bands over the intensity of full-length tRNA bands normalized to untreated controls is shown in (**F**). The relative expression of cleaved tRNA was quantified in three independent experiments (*t*-test, * *p* < 0.05).

It has been shown previously that exposure of cells to oxidative stress results in rapid deactivation of all tRNAs by specific endonucleolytic cleavage, which leads to the accumulation of tRNA-derived stress-induced RNA fragments (tiRNAs) [15,16]. Since the TRNT1-mediated addition of CCA to 3′ terminus of tRNAs protects them from degradation, we wanted to examine if, in SIFD-patient derived fibroblasts, there is an enhanced generation of tiRNA fragments. Control or SIFD patient-derived fibroblasts were treated with NaAsO_2_ for 1.5 or 18 h, and the generation of tiRNAs was examined by gel electrophoresis. We observed that in both treatment conditions, the SIFD patients exhibited markedly increased accumulation of tiRNAs (Figure 2A–D). To further determine if the enhanced tRNA cleavage is due to TRNT1 deficiency, we used siRNA to reduce levels of TRNT1 in C4 normal fibroblasts and subsequently treated these cells with NaAsO_2_ for 18 h. We observed that reducing levels of TRNT1 leads to a significant increase in the accumulation of tiRNAs in response to NaAsO_2_ treatment (Figure 2E,F).

### 3.3. Knock-down of Angiogenin Attenuates Cytotoxicity and tRNA Cleavage of SIFD Patient-Derived Cell during Oxidative Stress

**Figure 3:**
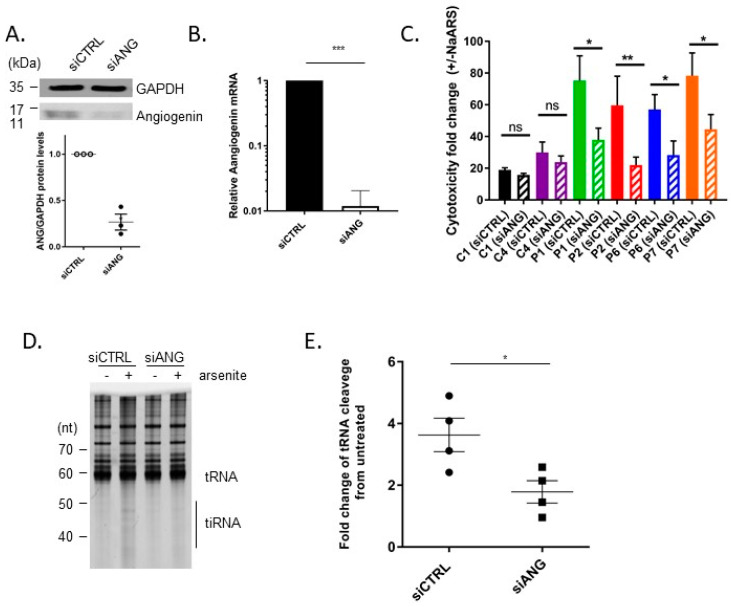
Angiogenin knock-down attenuates tRNA cleavage and sensitivity to oxidative stress in TRNT1 patient-derived cells. Normal (C1 and C4) and patient-derived fibroblasts (P1, P2, P6, P7) fibroblasts were transfected with angiogenin (siANG) or control (siCTRL) siRNA, and the relative levels of angiogenin and GAPDG (loading control) were determined by western blotting (**A**) or RT-qPCR (**B**) (C4 cells shown). For (**A**) representative western blot is shown on top; densitometric analysis of replicates is shown on the bottom (*n* = 3, *t*-test, *** *p* < 0.001). In a parallel experiment, siRNA transfected cells were subsequently treated with 50 μM NaAsO_2_, and the number of dead cells was monitored using the IncuCyte Live Imaging System and a DNA binding fluorescent reagent YOYO-1. The cytotoxicity fold change in NaAsO_2_ treated cells relative to non-treated cells was determined at 48 hrs for three independent experiments ((**C**) *t*-test, * *p* < 0.05, ** *p* < 0.01, ns—not significant). Patient-derived fibroblasts (P1) fibroblasts were transfected with angiogenin (siANG) or control (siCTRL) siRNA and subsequently treated with 50 μM NaAsO_2_ for 18 h. Total RNA was extracted and separated by gel electrophoresis as described in the Materials and Methods. A representative image is shown (**D**). The intensity of the cleaved tRNA bands over the intensity of full-length tRNA bands normalized to untreated controls is shown in (**E**). The relative expression of cleaved tRNA was quantified in four independent experiments. (*t*-test, * *p* < 0.05).

The ribonuclease responsible for tRNA cleavage in mammalian cells is angiogenin [17]. We, therefore, wished to determine if inhibiting angiogenin would protect SIFD patient-derived cells from arsenite-induced cytotoxicity and tRNA cleavage. Control and SIFD patient-derived fibroblasts were transiently transfected with siRNA-targeting angiogenin. The efficiency of knock-down was determined by western blotting (Figure 3A) and RT-qPCR (Figure 3B). Following angiogenin knock-down, the cells were treated with 50 μM NaAsO_2_ for 48 h, and the cytotoxicity was determined using YOYO-1 dye. We observed that reducing the levels of angiogenin partially rescued the sensitivity of SIFD patient-derived fibroblasts to oxidative stress (Figure 3C). To further determine the impact of angiogenin knock-down on tRNA cleavage, SIFD patient-derived P1 cells were transiently transfected with angiogenin-targeting siRNA, treated with 50 μM NaAsO_2_ for 18 h, and the accumulation of tiRNA was determined by gel electrophoresis. We observed that reducing angiogenin levels markedly decreased tiRNA levels (Figure 3D,E). 

### 3.4. TRNT1-Deficiency Results in Selective Changes in Protein Production

**Figure 4:**
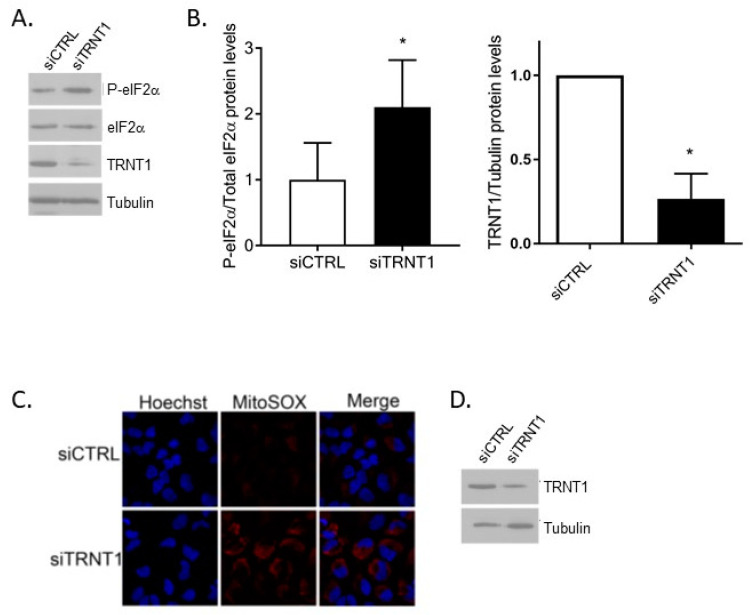
Reduction of TRNT1 or CCA1 levels results in increased phosphorylation of eIF2a and enhanced production of reactive oxygen species. (**A**) HeLa cells were transiently transfected with control or siRNA targeting *TRNT1*, and the levels of eIF2α, eIF2α-P, TRNT1, and tubulin (loading control) were determined by western blotting 48 h later. (**B**) The densitometry of three replicates is shown (*n* = 3, *t*-test, * *p* < 0.05). (**C**) HeLa cells treated as in (**A**) were labeled with MitoSox Red dye (a mitochondrial superoxide indicator) and visualized by microscopy (Hoechst (blue) = Nuclei). (**D**) The efficiency of *TRNT1* knock-down was confirmed by western blotting.

Accumulation of damaged or uncharged tRNAs in *TRNT1*-deficient cells would be expected to lead to activation of the eIF2α signaling axis. To examine this possibility, HeLa cells were transfected with control or *TRNT1*-targeting siRNA, and the phosphorylation of eIF2α was examined by western blotting after 48 h. We observed an approximately 2-fold increase in eIF2α phosphorylation (Figure 4A,B). In addition, acute reduction of TRNT1 leads to elevated levels of mitochondrial reactive oxygen species (Figure 4C,D).

**Figure 5:**
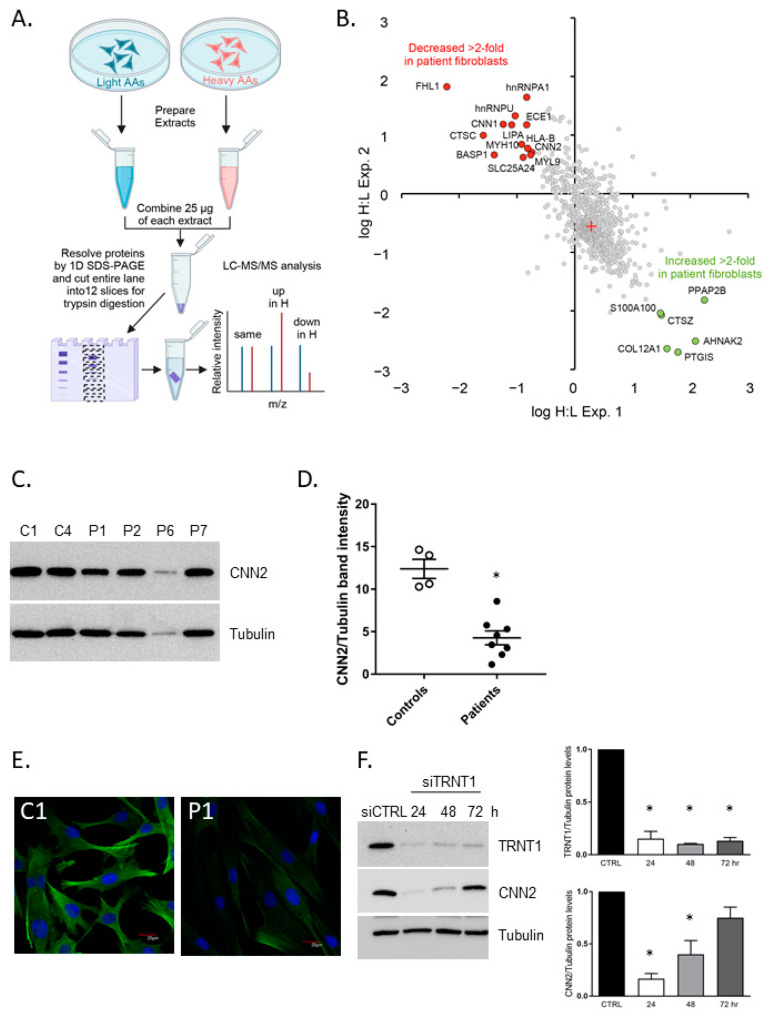
(**A**,**B**) Mass spectroscopy (SILAC) identification of differentially expressed proteins between patient (P1) and control (C1) fibroblasts. Reciprocal labeling (H:L) was used in experiments 1 and 2; up- and down-regulated proteins (shifted >2-fold from the mean) are highlighted, and the names are noted. The red cross represents the median log H:L (**C**) Expression of CNN2 was validated in a panel of control and SIFD patient-derived cells by western blotting. A representative blot is shown, and (**D**) the densitometry of repeated experiments. (**E**) The changes in CNN2 expression (green) were further examined in control (C1) and SIFD patient (P1) cells by fluorescent microscopy. Nuclei were stained with Hoeschst33342. (**F**) Control (C1) was transiently transfected with non-targeting (siCTRL) or *TRNT1*-targeting siRNA (siTRNT1), and the expression of CNN2 was determined 24, 48, and 72 h post-transfection by western blotting. Tubulin was used as a loading control. Representative western blots are shown on the left; densitometric analysis of replicates is shown on the right (*n* = 3, *t*-test, * *p* < 0.05).

Although phosphorylation of eIF2α is typically linked to general translational regulation, this was not observed in SIFD patient-derived fibroblasts [9]. However, smaller changes in eIF2α phosphorylation (such as those seen in cells with reduced levels of TRNT1, Figure 4A) could lead to altered selective translation of specific mRNAs (Clemens 2001). An inherited deficiency of TRNT1 enzyme activity would be expected to interfere with the normal maturation of both cytoplasmic and mitochondrial tRNAs and thus potentially negatively affect the translation of both nuclear- and mitochondrial-encoded proteins, including subunits of the respiratory chain complexes, as we have reported previously [9]. To examine this possibility in a more systematic and unbias manner, we used the Stable Isotope Labeling by Amino acids in Cell culture (SILAC) approach. Following isotope labeling, isolated mitochondria-enriched fractions of the cellular proteome from control (C1) and SIFD patient-derived fibroblasts (P1) were subjected to mass spectrometry analysis. We identified changes in the abundance of distinct groups of proteins (Figure 5A,B). Of the 25 proteins, GeneCard, OMIM, and NCBI were used to select proteins for further validation. These criteria included association with biological pathways, associated diseases, and links to phenotypes of SIFD. The availability of antibodies was determined through The Human Protein Atlas, and Antibodypedia was used to find suitable antibodies for each protein. The proteins CNN2, ECE1, CNN1, IGFBP7, FHL1, MYL9, ALDH1A1, and MIF were selected from the proteins expressed lower in the patient sample, while the IGFBP3, PTGIS, POSTN, and GAS6 were chosen from the proteins expressed higher in the patient sample. The antibodies for POSTN, IGFBP7, FHL1, and GAS6 did not identify the respective proteins in any of the lysate samples, so they were not used in any of the following western blot analyses. Thus, we have examined the expression of nine proteins (ALDH1A1, ECE1, MIF, IGFBP3, PTGIS, FHL1, MYL9, CNN1, and CNN2) in the panel of control (C1 and C4) and SIFD patient-derived fibroblasts (P1, P2, P6, and P7) by western blotting. Although the expression of some of these proteins differed between control and patient fibroblasts (e.g., CNN1, PTGIS, MIF), these differences were not statistically discernable (Supplementary Figure S3). In contrast, the expression of CNN2 was significantly decreased in all SIFD patient-derived fibroblasts (Figure 5C,D). Microscopic examination of P1 cells confirmed reduced expression of CNN2 when compared to C1 control fibroblasts (Figure 5E). In addition, transient, siRNA-mediated reduction of *TRNT1* levels in C1 control fibroblasts led to a marked decrease in CNN2.

## 4. Discussion

Transfer RNAs (tRNAs) play a central role in protein synthesis by translating genomic information in DNA into the amino acid sequences of protein [18,19]. More recently, it has been shown that tRNAs possess additional functions beyond involvement in protein synthesis, including involvement in cell stress response and the regulation of gene expression [18,20]. tRNA has also been identified as a source of a diversity of small noncoding RNA species through tRNA cleavage, generated by various mechanisms under varying stress and resting states in a large range of organisms [17,21]. tRNA cleavage is an evolutionarily conserved phenomenon, performing multifaceted functions across varying species [20,21]. The ribonucleases responsible for tRNA cleavage are angiogenin (ANG) in mammalian cells and Rny1 in yeast [17]. Under specific conditions, tRNA endures conformational changes, and nucleases gain access to mature tRNAs, cleaving the anticodon loop into 3′ and 5′ halves [21]. Therefore, this response generates an accumulation of tsRNAs, small fragments of RNA that are broadly classified into two types: tRNA-derived stress-induced RNA (tiRNA) and tRNA-derived fragments (tRFs), dependent on the location of the splicing site [16]. The complete functions of tRNA cleavage and its produced fragments are not entirely understood, and further research is needed to explore the potential cytotoxic qualities and roles in diseases. 

Oxidative stress is one of the triggers responsible for increasing tRNA cleavage and the levels of tRNA halves or ‘tiRNAs’ [22]. Under normal conditions, angiogenin remains inactive as it is bound by its inhibitor, ribonuclease/angiogenin inhibitor 1 (RNHI), and cytosolic tRNAs are not cleaved [23,24]. During oxidative stress, the levels of RNH1 protein decrease, leading to increased available free ANG, which cleaves tRNA, resulting in modifications at the structural and functional level [23,24,25,26]. Furthermore, the quantity of tiRNA is associated with the degree of cell damage induced by oxidative stressors, and the production of stress-induced tiRNA begins before evident cell damage or death [24]. This production of tiRNA leads to significant repression of cellular translation only in the presence of oxidative stress [27]. In addition to promoting the production of tiRNAs, the depletion of RNH1 also promotes CCA-deactivated tRNAs in cells [28], leading to the quick repression of translation during oxidative stress [15]. 

In addition to oxidative stress altering the conformational structure of tRNA from a whole into fragments, this process also alters tRNA at the functional level. tRNA halves likely have a complex range of effects, many of which are not yet fully understood [22]. Stress-induced tRNA cleavage-producing fragments have been thought to be a conserved feature of the cellular stress response and a cytoprotective mechanism that provides cell protection through the inhibition of translation and prevention of apoptosis [20,28]. However, recent evidence suggests the opposite, as tRNA plays a role in various diseases, and tiRNA has been shown to be cytotoxic or correlated with apoptosis initiations. For example, knocking out *RNH1* allowed ANG to accumulate in the cytosol, consequently promoting the cleavage and accumulation of tiRNA, several of them appearing to be cytotoxic and potential death inducers [29]. Similarly, in a study by Blanco et al. (2014), the accumulation of 5′tiRNA fragments as a result of ANG vulnerability due to hypomethylation of tRNA showed increased neuronal apoptosis, in addition to activation of cell stress pathways and translation repression [30]. Although the accumulation of specific 5′tiRNA fragments has been shown to be cytotoxic, the underlying complex regulatory mechanism is not fully understood [21]. In addition, oxidative stress was demonstrated to induce the down-regulation of tRNA methylase TRMT2A, and the consequence of this down-regulation is the enhancement of tRNA hypomodification, tsRNA formation, and ANG expression, and ultimately, disturbed tRNA stability [31]. 

The data presented in this study dovetail with the existing literature and help to explain cytotoxicity seen in SIFD patient cells. SIFD patient-associated mutations compromise the CCA-adding activity of TRNT1 [7,10], leaving pools of tRNAs susceptible to additional damage. Oxidative stress, both intrinsic and extrinsic, induces tRNA cleavage leading to enhanced cellular toxicity. In addition, changes in distinct tRNA abundance, as reported by Sasarman [10], combined with moderate eIF2α phosphorylation seen in cells with acute *TRNT1* reduction (Figure 4), would be expected to attenuate cellular protein synthesis of select proteins. Indeed, we show that levels of Calponin 2 are significantly reduced in all patient-derived cells following acute, siRNA-mediated depletion of TRNT1 (Figure 5). Calponin exists in three isoforms -1, 2, and 3. Calponin 2 (CNN2) is a 34kDa protein that binds calmodulin, actin, and tropomyosin to regulate smooth muscle contraction [32]. When bound to actin, CNN2 inhibits actin-activated myosin ATPase. It likely regulates actin filament stability and the levels of tropomyosin [33]. Notably relevant to the SIFD phenotype, CNN2 is found in various cell types, which contrasts with the other isoforms found mostly in smooth muscle and neuronal tissues [34]. CNN2 has also been investigated in relation to immune cell function, suggesting that a knock-down of *CNN2* in macrophages causes increased proliferation and migration [35]. We have also observed changes in levels of CNN1, although these were not statistically significant. Of note, CNN1 and CNN2 differ in their composition of certain amino acids; CNN2 has a higher amount of serine and aspartic acid residues, suggesting an attractive notion that these differences in amino acids could underpin the selective decrease of CNN2 in patient samples.

## 5. Conclusions

In this work, we show that *TRNT1* deficiency which accompanies SIFD, is associated with sensitivity to oxidative stress. This is due to exacerbated cleavage of tRNAs in an angiogenin-dependent manner. We further demonstrate that reduced levels of *TRNT1* lead to phosphorylation of eIF2α, increased ROS production, and changes in the abundance of distinct proteins. These observations suggest that the variable phenotypes that are seen in SIFD patients are likely due to dysregulation of tRNA maturation and abundance, which in turn negatively affects the translation of distinct proteins.

## Data Availability

The data presented in this study are available upon request from the corresponding author.

## Supplementary Materials

The following supporting information can be downloaded at: https://www.mdpi.com/article/10.3390/genes14051043/s1, Supplementary Figure S1: Induction of apoptosis by oxidative stress. Normal (C1) and patient-derived fibroblasts (P5, P6, and P7) were treated with hydrogen peroxide (A) or sodium arsenite (B), and the activation of caspases was measured using IncuCyte Live Imaging as described in material and methods. Supplementary Figure S2: Over-expression of *TRNT1* gene using a lentivirus-expressing vector in normal and SIFD patient-derived fibroblasts. Normal (C1) and patient-derived fibroblasts (P6 and P7) were transduced using a lentivirus vector expressing wild-type *TRNT1* or an empty vector. The transduction efficiency of the lentivirus in fibroblast was determined by detecting mCherry fluorescent signals using the Incucyte Live Imaging System (A). Western blot confirmed the expression of TRNT1 in normal and patient-derived fibroblasts. B-actin was used as a loading control (B). Representative western blots are shown on the left; densitometric analysis of replicates is shown on the right (*n* = 3, *t*-test, * *p* < 0.05). Supplementary Figure S3: Validation of protein targets identified by SILAC. Normal (C1 and C4) and patient-derived fibroblasts (P1, P2, P6, P7) fibroblasts were grown to confluency, and the levels of ALDH1A1, ECE1, MIF, IGFBP3, PTGIS, FHL1, MYL9, CNN1, and tubulin (loading control) were determined by western blotting. Densitometry of 3–4 replicates is shown.
