# Supplementary material for: TRNT-1 Deficiency Is Associated with Loss of tRNA Integrity and Imbalance of Distinct Proteins
Source: Genes (Basel). 2023 May 5;14(5):1043. doi: 10.3390/genes14051043 (PMC10218503; doi:10.3390/genes14051043)
Supplement: Supplementary file 1 [file genes-14-01043-s001.zip › genes-2339212-supplementary.pdf]

Supplementary Figure S1

A.

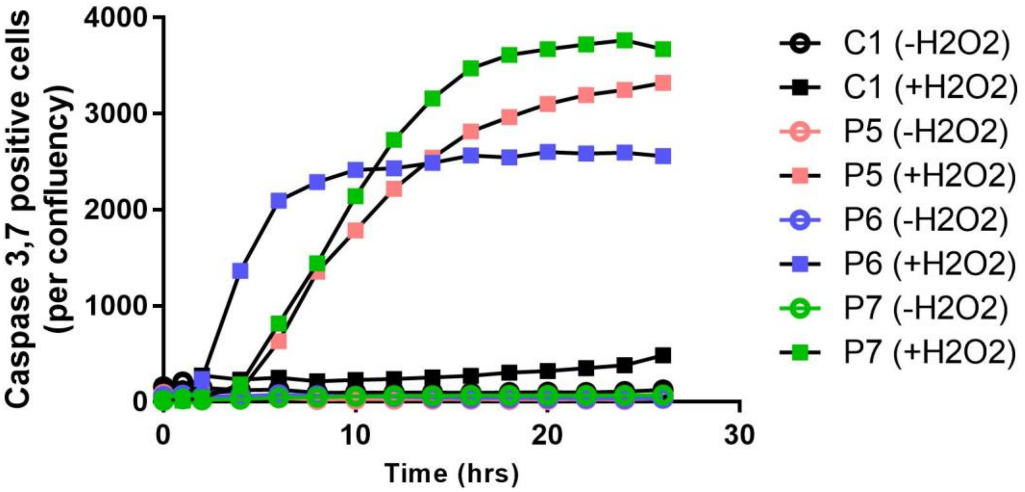

B.

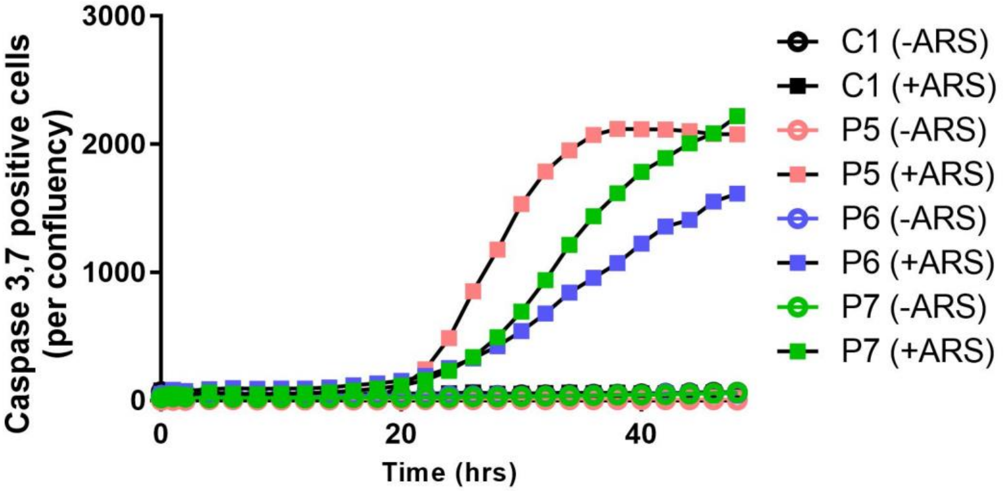

Supplementary Figure S1. Induction of apoptosis by oxidative stress. Normal (C1) and patient-derived fibroblasts (P5, P6, and P7) were treated with hydrogen peroxide (A) or sodium arsenite (B), and the activation of caspases was measured using IncuCyte Live Imaging as described in material and methods.

Supplementary Figure S2

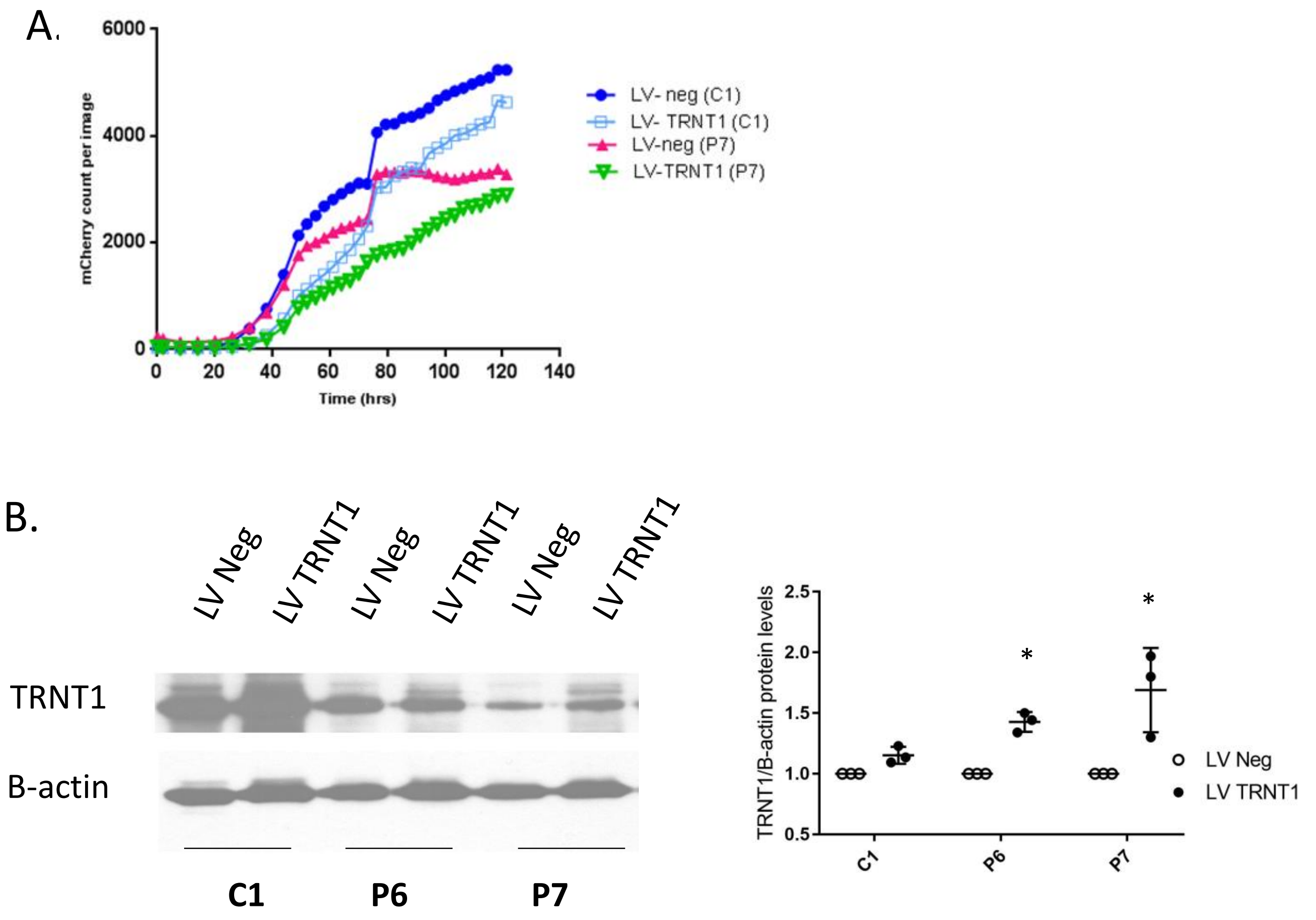

Supplementary Figure S2. Over-expression of *TRNT1* gene using a lentivirus-expressing vector in normal and SIFD patient-derived fibroblasts. Normal (C1) and patient-derived fibroblasts (P6 and P7) were transduced using a lentivirus vector expressing wild-type *TRNT1* or an empty vector. The transduction efficiency of the lentivirus in fibroblast was determined by detecting mCherry fluorescent signals using the Incucyte Live Imaging System (A). Western blot confirmed the expression of TRNT1 in normal and patient-derived fibroblasts. B-actin was used as a loading control (B). Representative western blots are shown on the left; densitometric analysis of replicates is shown on the right (n = 3, t-test, * $p < 0.05$).

Supplementary Figure S3

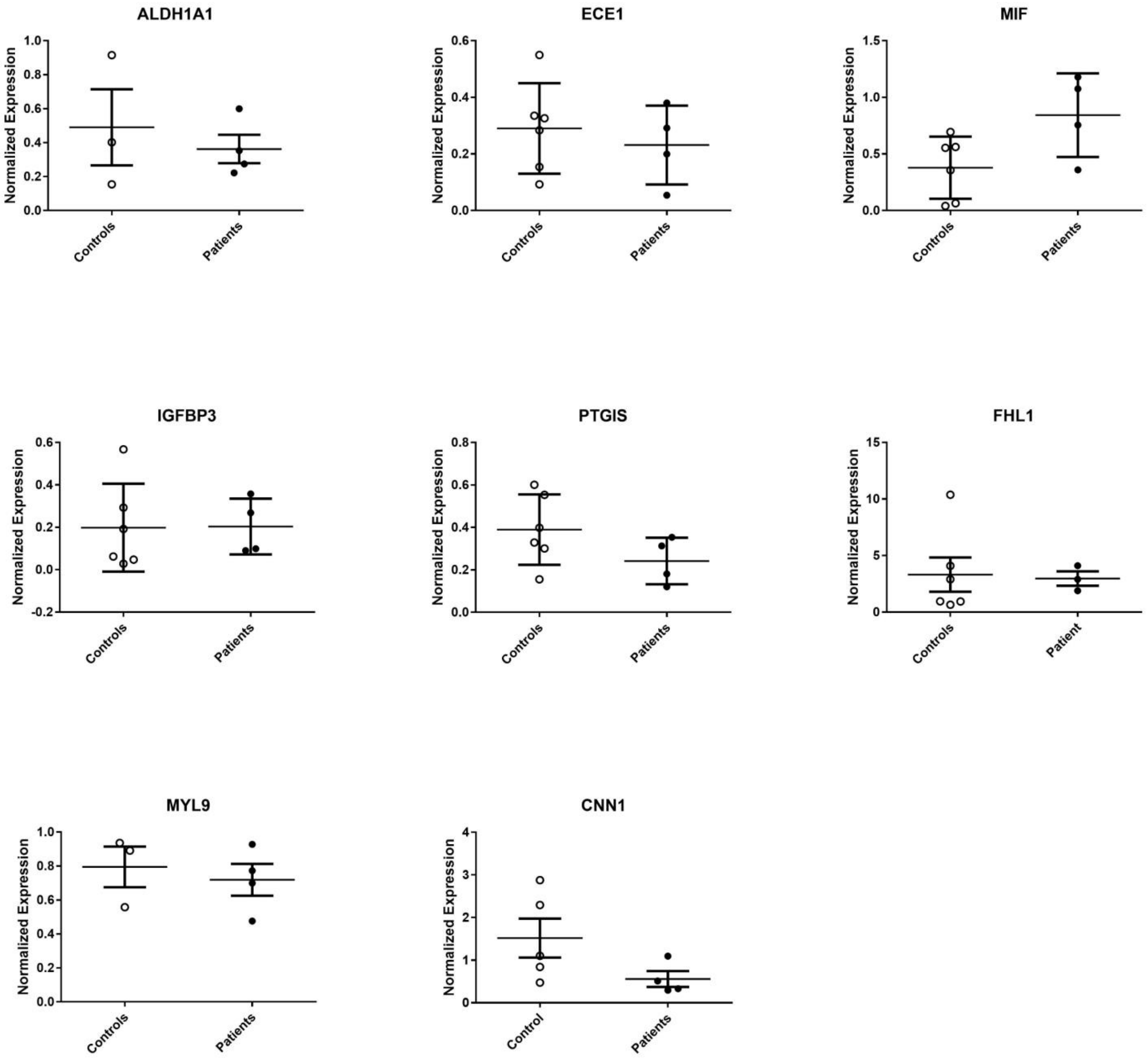

Supplementary Figure S3. Validation of protein targets identified by SILAC. Normal (C1 and C4) and patient-derived fibroblasts (P1, P2, P6, P7) fibroblasts were grown to confluency, and the levels of ALDH1A1, ECE1, MIF, IGFBP3, PTGIS, FHL1, MYL9, CNN1, and tubulin (loading control) were determined by western blotting. Densitometry of 3–4 replicates is shown.
